# Corazonin Stimulates Ecdysteroid Synthesis during the Molting Process of the Swimming Crab, *Portunus trituberculatus*

**DOI:** 10.3390/biology13080630

**Published:** 2024-08-18

**Authors:** Xi Xie, Jun Zhang, Shisheng Tu, Qi Zhou, Dongfa Zhu

**Affiliations:** Key Laboratory of Aquacultural Biotechnology Ministry of Education, School of Marine Sciences, Ningbo University, Ningbo 315832, China; xiexi@nbu.edu.cn (X.X.);

**Keywords:** corazonin, corazonin receptor, *Portunus trituberculatus*, ecdysteroid synthesis, molting

## Abstract

**Simple Summary:**

Functional studies of neuropeptides are of great significance in revealing the neuroendocrine regulation mechanism in crustaceans, and in guiding and developing artificial regulation techniques in economic species. In this paper, we investigated the effect of a crustacean neuropeptide corazonin (Crz) on ecdysteroid synthesis based on the high expression of Crz receptor in the Y-organ, the main site of ecdysteroid synthesis. By using Crz treatment and CrzR silencing, we found that Crz/CrzR signaling could cause up-regulation of ecdysteroid synthesis genes and an increase in ecdysteroid levels both in vitro and in vivo. In addition, it was also observed that Crz/CrzR signaling affects the expression level of ecdysis-triggering hormones. Our results provide new insights into the understanding of Crz function in crustaceans.

**Abstract:**

The neuropeptide corazonin (Crz) exerts diverse physiological effects in insects, yet its role in crustaceans remains elusive. The abundant expression of Crz receptor (CrzR) in the Y-organs of several crustaceans suggests a potential involvement of Crz in regulating ecdysteroid synthesis. In this study, we examined the effects of PtCrz on ecdysteroid synthesis during the molting period of *Portunus trituberculatus* through PtCrz treatments and PtCrzR silencing. Our results showed that PtCrz peptide stimulates ecdysteroid levels and the gene expression involved in ecdysteroidogenesis both in vitro and in vivo, whereas dsPtCrzR treatments had opposite effects on ecdysteroid levels and associated gene expression. Thus, our study suggests that PtCrz may modulate ecdysteroid synthesis via Y-organ-expressed PtCrzR. Furthermore, we also discovered the involvement of PtCrz/PtCrzR signaling in regulating *PtETH* expression. Notably, the inhibitory effect of dsPtCrzR on ecdysteroid synthesis or *PtETH* expression can be reversed by PtCrz treatment, suggesting the potential existence of multiple receptors for PtCrz. This study provides new insights into the function of crustacean Crz and, for the first time, elucidates the presence of a neuropeptide that can stimulate ecdysteroid synthesis in crustaceans.

## 1. Introduction

Corazonin (Crz) is a peptidergic neurohormone widely distributed among arthropods. Initially discovered as a cardioacceleratory peptide in the cockroach *Periplaneta americana* [1], its function as such is not universally observed across all species [2,3]. Crz may also play a role in nutritional stress signaling [4], behavior modulation [5,6,7], and reproductive physiology [6,8,9] among other functions, which may vary among species. All Crz prepropeptides contain a predicted signal peptide, a conserved mature peptide of 11 amino acids, and an additional sequence known as Crz-precursor-related peptide (CrzRP) [10,11]. Among the six isoforms of mature Crzs, [Arg7]-corazonin is the most common form found in insects [12] and the sole form present in crustaceans [13].

The diverse physiological effects of Crz are primarily mediated by its membrane receptor (CrzR), a member of the G-protein coupled receptor (GPCR) family. In *Drosophila melanogaster*, gene silencing of CrzR often leads to altered physiological states, including changes in stress responses, feeding behavior, and energy metabolism; all of these phenotypes are associated with Crz action [14,15]. Structurally homologous to the mammalian gonadotropin-releasing hormone (GnRH) receptor [16], CrzR has been identified and characterized in various insects [5,17,18,19,20,21]. Upon activation, CrzR releases secondary messengers such as cAMP and Ca^2+^, thereby regulating cellular physiological functions [19]. The tissue distribution of CrzR also varies among different species, which may reflect the variable functions of Crz. Notably, in the tobacco hornworm *Manduca sexta*, the high expression of CrzR in Inka cells is crucial for initiating ecdysis as its activation by Crz leads to the release of ecdysis-triggering hormone (ETH) [5].

In recent years, advances in transcriptomics have facilitated the identification of Crz and CrzR sequences across various crustacean species [3,22,23,24,25]. However, a comprehensive understanding of the Crz/CrzR signaling functions within crustaceans remains incomplete. Crz had no effect on heart activity, blood glucose levels, lipid mobilization, or pigment distribution in chromatophores in the green shore crab *Carcinus maenas* [3]. By contrast, it influences pigment migration in the crayfish *Procambarus clarkia* [26], and some molt behavior in the redclaw crayfish *Cherax quadricarinatus* [27]. In a previous investigation, we discovered that the Crz/CrzR signaling system may also be involved in the vitellogenesis process of *Portunus trituberculatus*, thereby uncovering a novel function for this pathway [10]. It is noteworthy that that study not only revealed that *P. trituberculatus* Crz is predominantly derived from neural tissue, but also discovered a high expression of *CrzR* in the Y-organ, a primary site for crustacean ecdysteroid biosynthesis [10]. Extremely high *CrzR* levels were also observed in *C. maenas* Y-organ; however, it is paradoxical that while application of the Crz peptide had no significant impact on ecdysteroid biosynthesis in *C. maena* (with exception of slight stimulation during early postmolt) [3], it did induce an increase in expression of several ecdysteroidogenesis genes in our cultured Y-organ explant [10].

Although it is common for the Crz/CrzR action to vary across species, our aforementioned studies were specifically conducted during the period of ovarian development. In this current study, we utilized both in vitro and in vivo Crz treatments, as well as CrzR RNA interference (RNAi), to investigate whether Crz/CrzR signaling stimulates ecdysteroid synthesis during the molting period. Furthermore, given the findings in *Manduca sexta* and *Cherax quadricarinatus* indicating potential involvement of Crz/CrzR signaling in ETH action [5,27], we further explored their potential regulatory role on *ETH* expression.

## 2. Materials and Methods

### 2.1. Experimental Animals

Juvenile male crabs (40–70 g of bodyweight) were purchased from Xianxiang, Ningbo, in August 2021 and reared at the aquaculture base of the Institute of Marine and Fisheries, Ningbo, China. The crabs were kept in large tanks with continuous seawater flow and fed live razor clams once a day. Different stages of the molt cycle, including postmolt (stages A and B), intermolt (stage C), and premolt (stage D_0_, D_1_, D_2_, D_3_, and D_4_), were determined by observing the hardness of the carapace and the formation of new epidermis as described previously [28]. All crabs were anaesthetized on ice for 10 min prior to sacrifice, and tissues including the Y-organ, eyestalk, brain, and thoracic ganglion were dissected and stored in RNA preservation fluid (Cwbiotech, Taizhou, China) at −80 °C until RNA extraction.

### 2.2. In Vitro Treatments

The mature PtCrz peptide (pQTFQYSRGWTN-NH2) was synthesized by Sangon Biotech (Shanghai, China), with a purity of 98%. The cDNA fragments of the PtCrzR transmembrane region (565 bp) and the green fluorescent protein (GFP, 568 bp) were cloned into the pMD19-T vector (Takara, Kyoto, Japan), respectively, and the corresponding dsRNAs were prepared as previously described [10]. Since the hemolymph ecdysteroid titer generally begins to rise during the premolt stages during a natural molt, Y-organs from premolt D_0_ crabs were utilized for in vitro experiments. For PtCrz treatments, Y-organ explants were exposed to synthetic PtCrz peptide at final concentrations of 10^−5^ M, 10^−6^ M, and 10^−7^ M after pre-incubation for 1 h. The control group received an equivalent volume of crab saline. In the case of dsPtCrzR treatments, Y-organ explants from the same stage were treated with dsPtCrzR, dsGFP, and dsCrzR supplemented with PtCrz at 10^−6^ M (the dosage was chosen according to the results of the above PtCrz treatment experiments). The dosage of dsRNA was set at 5 μg per plate. Following an additional incubation period of 8 h, all tissue explants and corresponding culture mediums were collected for further analysis of gene expression and ecdysteroid levels.

### 2.3. In Vivo Treatments

Crabs in the premolt stage at D_0_ and D_2_ were selected for PtCrz in vivo injection. Considering that hemolymph volumes are estimated to be approximately 27% of body mass [29], the dosage of PtCrz injection (3–4.2 × 10^−4^ M in 50 μL) was calculated to maintain a hemolymph concentration of added Crz at 10^−6^ M. The control group received an equivalent volume of crab saline. Crabs at premolt D_0_ were utilized for in vivo RNA interference, with groups corresponding to those used in the in vitro experiments, including dsGFP, dsPtCrzR, and dsPtCrzR + Crz groups. The dosage of dsRNA was standardized at 3 μg/g bodyweight. All injections were administered at the base of the last walking foot using a 1 mL syringe. Hemolymph and tissue samples were collected 24 h post-injection for further analysis of gene expression and ecdysteroid levels.

### 2.4. Determination of Ecdysteroid Levels

The ecdysteroid levels in culture medium and hemolymph samples were quantified using the 20-Hydroxyecdysone Enzyme Immunoassay Kit (ARBOR ASSAYS, Ann Arbor, MI, USA). Briefly, 0.5 mL of culture medium and hemolymph was extracted, added to 1.5 mL of methanol, vortexed thoroughly, and then centrifuged at 4 °C for 15 min at 12,000× *g*. The resulting supernatant was transferred to a new sterile centrifuge tube, dried using a nitrogen blower, and stored at −80 °C. Prior to use, the dried powder was reconstituted in diluted Assay Buffer (1:5 with sterile water) and analyzed according to the provided kit protocols.

### 2.5. Gene Expression Analysis

The relative gene expression levels were determined using quantitative reverse-transcription PCR (qRT-PCR) with specific primers (Table 1). The qRT-PCR was performed on the ABI 7500 qPCR instrument (Thermofisher, Waltham, MA, USA) following the manufacturer’s instructions of the SYBR^®^ Premix Ex Taq™ II Kit (Takara). PCR conditions included an initial denaturation at 95 °C for 2 min, followed by 40 cycles of amplification at 95 °C for 15 s and extension at 56 °C for 20 s. Additional melting curve analysis was conducted to confirm product specificity, with temperature increasing from 55 to 95 °C at a rate of 0.2 °C/s. Amplification efficiencies were assessed using standard curve analysis based on a five-point, tenfold dilution series of cDNA. Each sample was analyzed in triplicate for technical replicates. Normalization of target gene expression was achieved using β-actin, and relative mRNA expression levels were calculated utilizing the comparative Ct (2^−ΔΔCt^) method [30].

### 2.6. Statistical Analysis

The data were subjected to normality testing using the Kolmogorov–Smirnov and Cochran tests prior to all statistical analyses. Non-normally distributed data were assessed using the nonparametric Mann–Whitney test, while normally distributed data were analyzed using one-way ANOVA followed by Tukey’s test or the student’s *t*-test (SPSS 24.0 software, IBM, Armonk, NY, USA). Statistical significance was considered at *p* < 0.05 and denoted with letters or asterisks in all cases.

## 3. Results

### 3.1. Expression Profiles of PtCrz and PtCrzR during the Molt Cycle

**Figure 1:**
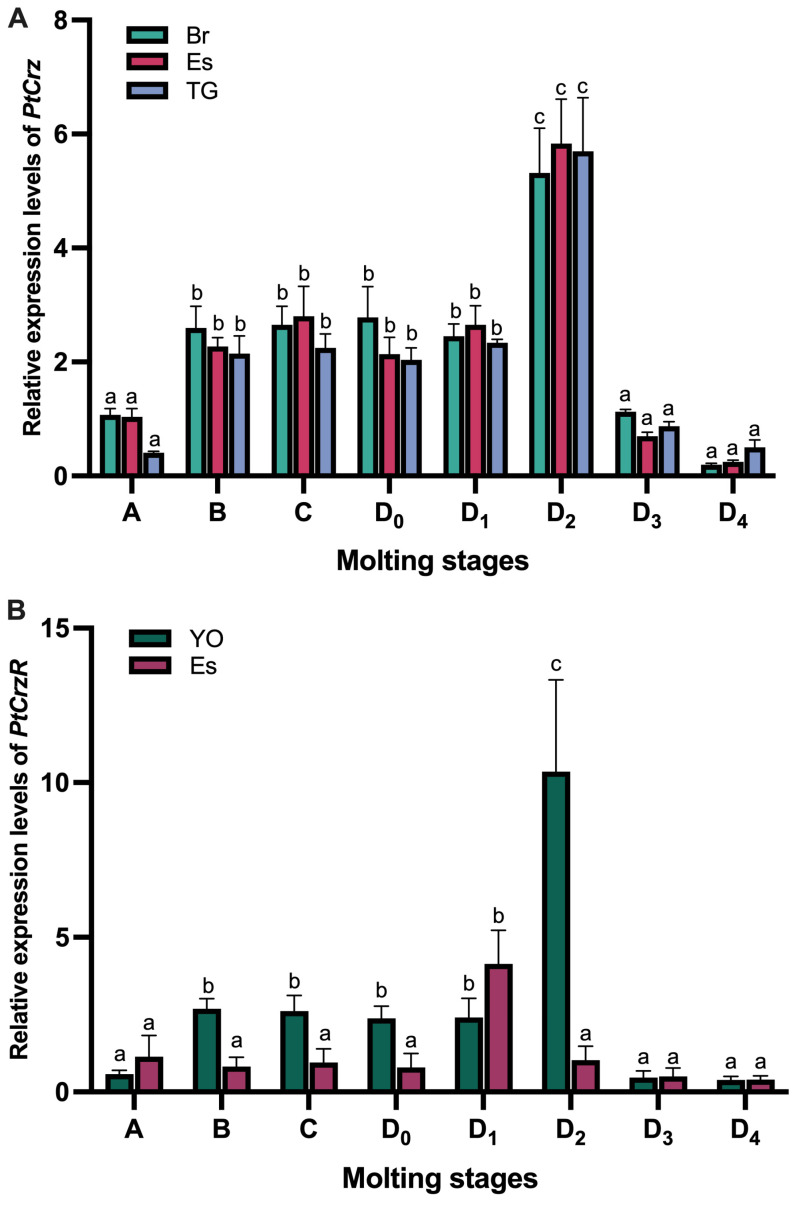
Expression profiles of PtCrz (**A**) and PtCrzR (**B**) during the molt cycle of *P. trituberculatus.* The relative mRNA levels of PtCrz were examined in brain (Br), eyestalk (Es), and thoracic ganglion (TG), while PtCrzR were examined in Y-organ (YO) and eyestalk (Es). The molting stages includes postmolt (stage A and B), intermolt (stage C), and premolt (stage D_0_, D_1_, D_2_, D_3_, and D_4_). Bars represent mean + SEM (n = 5). Different letters indicate statistically significant differences within each tissue (*p* < 0.05).

Based on previous reports of tissue distribution [10], we conducted an analysis of the variations in *PtCrz* expression levels in the eyestalk, brain, and thoracic ganglion, as well as *PtCrzR* expression in the Y-organ and eyestalk throughout the molt cycle. While the expression patterns of *PtCrz* in these three nerve tissues were not identical, they all exhibited a gradual increase from postmolt (stage A), reaching a peak at premolt D_2_ substage, followed by a rapid decline to a minimum at premolt D_4_ (Figure 1A). A similar trend was observed for *PtCrzR* expression in the Y-organ; however, the peak of *PtCrzR* expression in the eyestalk occurred at premolt D_1_ (Figure 1B).

### 3.2. Effects of PtCrz Peptide and dsPtCrzR on Ecdysteroid Synthesis In Vitro

**Figure 2:**
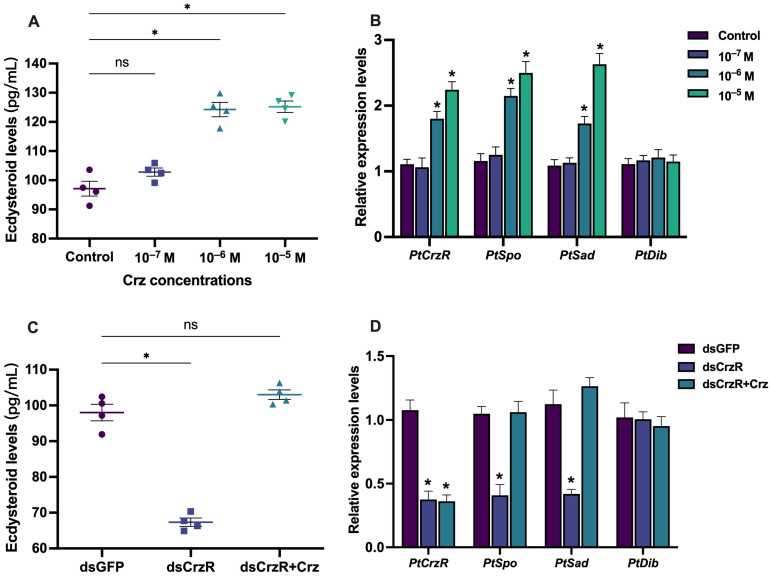
Effects of PtCrz peptide and dsPtCrzR on ecdysteroid synthesis in vitro. The ecdysteroid levels in Y-organ culture medium (**A**) and the *PtCrzR*, *PtSpo*, *PtSad*, and *PtDib* expression levels (**B**) when treated with different concentrations of Crz peptides. The ecdysteroid levels in Y-organ culture medium (**C**) and the *PtCrzR*, *PtSpo*, *PtSad*, and *PtDib* expression levels (**D**) when treated with different dsGFP, dsCrzR, and dsCrzR supplemented with PtCrz at 10^–6^ M. Bars represent mean ± SEM (n = 4). The asterisk indicates a statistically significant difference when compared with control or dsGFP group (*p* < 0.05), while “ns” means no significant difference.

We investigated the effects of varying concentrations (10^−7^ M, 10^−6^ M, and 10^−5^ M) of PtCrz peptide on ecdysteroid synthesis in the Y-organ cultured in vitro. Our findings revealed that exposure to 10^−6^ M and 10^−5^ M PtCrz resulted in significantly elevated levels of ecdysteroids (124.2 ± 2.5 pg/mL and 125.2 ± 2.0 pg/mL, respectively) in the culture medium compared to the control group (97.1 + 2.5 pg/mL) (Figure 2A). By contrast, the ecdysteroids levels in 10^−7^ M PtCrz treatment (102.8 + 1.4 pg/mL) showed no significant difference with the control group. Furthermore, these treatments effectively stimulated the expression of *PtCrzR* and induced the expression of two ecdysteroid synthesis pathway genes (*PtSpo* and *PtSad*), while showing no discernible effect on another ecdysteroidogenesis gene, *PtDib* (Figure 2B). On the other hand, treatment with dsPtCrzR led to a marked decrease in *PtCrzR* within the Y-organ, resulting in opposing effects on ecdysteroid levels (98.0 ± 2.3 pg/mL for dsGFP and 67.3 ± 1.2 pg/mL for dsCrzR) and *PtSpo* and *PtSad* expression compared to PtCrz treatments (Figure 2C,D). However, it was observed that such effects could be recovered by administration of PtCrz peptide.

### 3.3. Effects of PtCrz Peptide and dsPtCrzR on Ecdysteroid Synthesis In Vivo

**Figure 3:**
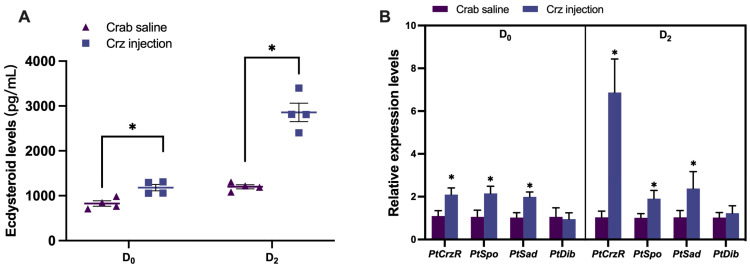
Effects of PtCrz peptide injection on ecdysteroid synthesis in vivo. (**A**) Induced hemolymph ecdysteroid levels by injection with PtCrz peptide in premolt D_0_ and D_2_, respectively. (**B**) The change in expression levels of *PtCrzR*, *PtSpo*, *PtSad*, and *PtDib* in Y-organ after PtCrz peptide treatments in two stages. Bars represent mean ± SEM (n = 4). The asterisk indicates a statistically significant difference when compared with crab saline group (*p* < 0.05).

**Figure 4:**
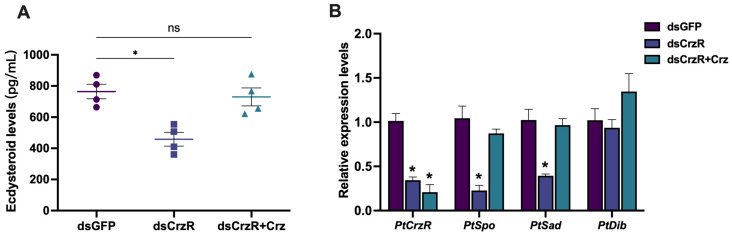
Effects of dsPtCrzR on ecdysteroid synthesis in vivo. The hemolymph ecdysteroid levels (**A**) and the *PtCrzR*, *PtSpo*, *PtSad*, and *PtDib* expression levels (**B**) when treated with different dsGFP, dsCrzR, and dsCrzR supplemented with PtCrz at 10^−6^ M. Bars represent mean ± SEM (n = 4). The asterisk indicates statistically significant different when compared with control group (*p* < 0.05), while “ns” means no significant difference.

Similar to the findings observed in in vitro experiments, PtCrz peptide was found to stimulate hemolymph ecdysteroid levels (Figure 3A). The hemolymph ecdysteroid titer increased from 827.5 ± 120.6 pg/mL of the crab saline group to 1179.0 ± 141.5 pg/mL of the Crz injection group in the premolt D_0_ stage, and from 1198.8 ± 92.3 pg/mL to 2855.7 ± 409.9 pg/mL in the premolt D_2_ stage. In addition, the expression of *PtCrzR*, *PtSpo*, and *PtSad* during both premolt D_0_ and premolt D_2_ stages were also upregulated after Crz treatment (Figure 3B). Notably, the upregulation of PtCrzR was more pronounced during premolt D_2_, exhibiting an approximately 6-fold increase compared to the crab saline group, while only showing an approximately 2-fold increase during premolt D_0_. Treatment with dsPtCrzR in premolt D_0_ resulted in contrasting effects on ecdysteroid levels (764.4 ± 93.0 pg/mL for dsGFP and 457.9 ± 88.4 pg/mL for dsCrzR) and associated gene expression, which could be rescued by administration of PtCrz (Figure 4).

### 3.4. Effects of PtCrz/PtCrzR Signaling on PtETH Expression

**Figure 5:**
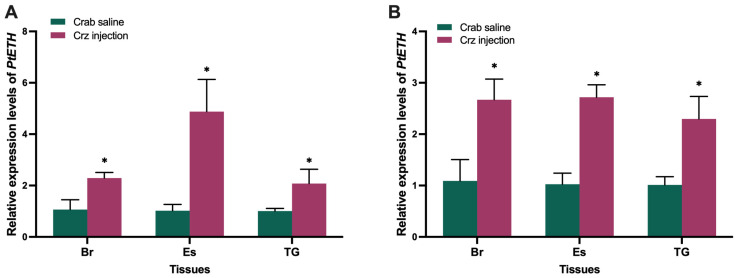
Effects of PtCrz peptide on *PtETH* expression. The *PtETH* expression was examined in brain (Br), eyestalk (Es), and thoracic ganglion (TG) in treatments including PtCrz injection in premolt D_0_ (**A**) and premolt D_2_ (**B**). Bars represent mean ± SEM (n = 4). The asterisk indicates a statistically significant difference when compared with crab saline group (*p* < 0.05).

**Figure 6:**
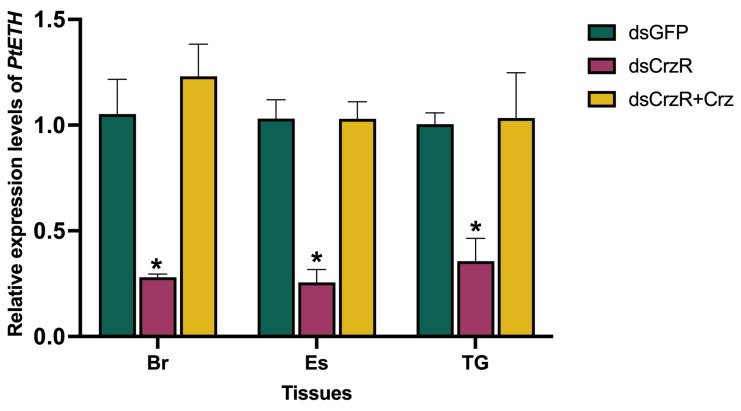
Effects of dsPtCrzR injection on *PtETH* expression. The *PtETH* expression was examined in brain (Br), eyestalk (Es), and thoracic ganglion (TG). Bars represent mean ± SEM (n = 4). The asterisk indicates a statistically significant difference when compared with dsGFP group (*p* < 0.05).

The expression of *PtETH* in nerve tissues, including the brain, eyestalk, and thoracic ganglion, was detected in the aforementioned in vivo experiments. It was observed that *PtETH* expression in these three tissues was upregulated by PtCrz peptide (Figure 5) and downregulated by dsPtCrzR (Figure 6). Furthermore, the inhibitory effects of dsPtCrzR could be rescued by PtCrz injection.

## 4. Discussion

In this study, we investigated the effect of PtCrz on ecdysteroid synthesis during the molting period of *P. trituberculatus* through neuropeptide treatments and RNA silencing of its receptor. Combined with our previous research [10], we suggest that PtCrz plays a regulatory role in ecdysteroidogenesis in *P. trituberculatus*, both during ovarian development and molting process. While our results indicate the involvement of PtCrzR in mediating the effects of PtCrz, it is plausible to consider the potential involvement of other Crz receptors, as evidenced by the recovery of dsPtCrzR effects following PtCrz treatment. Notably, there are two types of CrzRs expressed in the Y-organ of the blackback crab *Gecarcinus lateralis*, belonging to GPCR A6 and A7, respectively [24]. Further phylogenetic analysis showed that PtCrzR is more closely related to GPCR A6 type (Supplementary Material), while the A7 type of CrzR was not found in our transcriptomic data. Certainly, given that the dsRNA utilized in this investigation is designed for gene knockdown rather than gene knockout, it is also plausible that the impact of Crz may not be fully attenuated. Consequently, further research is warranted to validate the aforementioned hypothesis.

Our findings are in contrast with the observed effects of Crz in the green shore crab, *C. maenas*, where the Crz peptide only weakly stimulated ecdysteroid synthesis during early postmolt [3]. As previously mentioned, the function of the Crz peptide may vary between species. However, this inconsistency is intriguing considering that CrzR was highly expressed in the Y-organ of both species and that both PtCrzR and CmCrzR clustered together in phylogenetic analysis (Supplementary Material). It should be noted that the concentration of Crz administered in our study was substantially higher (10^−6^ M in this study vs. 5 × 10^−8^ M in *C. maenas*), but both levels exceeded their respective EC_50_ values for CrzR activation in each species. Thus, the discrepancy cannot be readily attributed to the amount of Crz applied.

Instead, the variation in responses may be attributed to intrinsic functional disparities within the receptors themselves. For instance, PtCrzR is clustered together with GlCrzR-A6, but their expression patterns differ throughout the molt cycle: *PtCrzR* is significantly upregulated in premolt D_2_, while *GlCrzRA6* shows high expression only in postmolt stages [24], indicating potential divergent functional roles for the same type of CrzR across species. In fact, although not examined, the expression of *CmCrzR* is high, at least in the intermolt, the period from which its tissue distribution was analyzed [3]. This is very different from the expression pattern of *GlCrzRA6* and possibly also *PtCrzR*. On the other hand, the expressions of *PtCrz* and *PtCrzR* during the molt cycle exhibit a strong correlation with ecdysteroidogenesis, as hormone levels peak in premolt D_3_ [31] following the peak expressions of *PtCrz* and *PtCrzR*.

To the best of our knowledge, this study represents the first documentation of a neuropeptide’s involvement in promoting ecdysteroid synthesis in crustaceans. PtCrz may work in conjunction with classical molt-inhibiting hormone (MIH), which belongs to the crustacean hyperglycemic hormone (CHH) family of neuropeptides [32]. It is generally accepted that MIH signaling suppresses ecdysteroidogenesis through the cyclic nucleotide second messengers cAMP and cGMP [33]. Therefore, PtCrz/PtCrzR may potentially utilize alternative GPCR mediated pathways, such as the phospholipase C (PLC)/PKC pathway, which has been suggested to promote ecdysteroid synthesis and secretion by the Y-organ [11]. The decrease in hemolymph levels of MIH during late intermolt triggers ecdysteroid synthesis in the Y-organ, while high levels of MIH reappear during mid-premolt, where it is no longer sensitive to the Y-organ [33]. In contrast, the Y-organ appears to be more sensitive to PtCrz during premolt D_2_, as evidenced by PtCrz inducing greater fold changes in PtCrzR expression and ecdysteroid levels compared to premolt D_0_. However, given the expression of multiple neuropeptide receptors in the crustacean Y-organ [24,34], it is still open for discussion whether Crz serves as the primary regulator in stimulating ecdysteroidogenesis. It is worth noting that ecdysteroid synthesis in Drosophila is governed by a complex network of multiple neuropeptides [35], and a similar scenario can be anticipated in crustaceans.

In order to stimulate ecdysteroid levels, the Crz signaling system may target the genes that encode enzymes involved in the ecdysteroid biosynthetic pathway. This is supported by the altered expression of two such genes, *PtSpo* and *PtSad*, in both Crz treatment and CrzR RNAi. The classical ecdysteroid biosynthesis pathway involves seven enzymes, five of which are cytochrome P450 monooxygenases (CYPs). Their coding genes are collectively known as the Halloween genes, including neverland (nvd), non-molting glossy/shroud (sro), spook (spo, CYP307A1), phantom (phm, CYP306A1), disembodied (dib, CYP302A1), shadow (sad: CYP315A1), and shade (shd: CYP314A1) [36]. *PtSpo*, *PtDib*, and *PtSad* were selected for expression analysis in this study because their dsRNA can downregulate hemolymph ecdysteroid levels [37]. In contrast to *PtSpo* and *PtSad*, *PtDib* expression was not sensitive to Crz treatment or CrzR RNAi, suggesting that Crz/CrzR signaling only targets certain Halloween genes. Interestingly, eyestalk ablation, a common method to induce hemolymph ecdysteroid levels, induces the expression of *PtDib* and *PtSad* but not *PtSpo* [37]. This again suggests a complex neuropeptide system in the control of crustacean ecdysteroid synthesis.

In the tobacco hornworm *Manduca sexta*, Crz was able to stimulate early secretion of the ecdysis-triggering hormone (ETH), which in turn initiated the onset of the ecdysis behavioral sequence [5]. Crz may play a similar role in crustaceans, as demonstrated in the redclaw crayfish *Cherax quadricarinatus*, where Crz injection induced typical molting responses, albeit in a slower and more asynchronous manner than that observed with ETH injection. Our results showed that Crz treatments at two premolt stages significantly promoted *PtETH* expression in three neural tissues, suggesting a potential function in controlling ETH action. However, neither PtCrz nor PtETH induced any putative molting responses in vivo (data is being prepared and scheduled for future published). Therefore, the physiological significance of this result remains to be investigated. It has recently been proposed that ETH may possess functions beyond behavioral regulation, as evidenced by its RNA silencing in the mud crab *Scylla paramamosain* leading to widespread mortality and perturbations in numerous metabolic pathways-related genes [38,39].

## 5. Conclusions

In conclusion, the current study demonstrated that PtCrz plays a stimulatory role in ecdysteroid synthesis and may be involved in the regulation of PtETH expression. CrzR is involved in both actions of Crz, but may not be the only Crz receptor. Our results provide new insights into the understanding of Crz function in crustaceans.

## Figures and Tables

**Table 1:** The qRT-PCR primers used in this study.

Gene	Primer (5′-3′)	GenBank Accession No.
PtCrz	Forward: CAGTTGTGGTGCTCGTTGCC	OL694705
	Reverse: GCTCAGCGGACCTTTTTCG	
PtCrzR	Forward: GTCATCTGCTGGACTCCCTACTAC	OL694706
	Reverse: CGGGTTGACGAGACTGTTGG	
PtSpo	Forward: GTTTTGGCTCCCGCAACTA	KM030021
	Reverse: TGTCGTCGGTGAGGCTTGT	
PtSad	Forward: CAGATATGGGCAGATTCATCG	KM596851
	Reverse: AAGGCGTCATCCAGGCAC	
PtDib	Forward: GGCAAACACTGGTGGGAACT	KM880023
	Reverse: ACCCTTCACGCCTCATCTTG	
PtETH	Forward: ATGCTCTCTGTTCTGGACTCAAG	MT890695
	Reverse: TCACTTCTGCAGGTAACGCA	
β-actin	Forward: CGAAACCTTCAACACTCCCG	FI641977
	Reverse: CGGGTTGACGAGACTGTTGG	

## Data Availability

The data that support the findings of this study are available from the corresponding author upon reasonable request.

## Supplementary Materials

The following supporting information can be downloaded at: https://www.mdpi.com/article/10.3390/biology13080630/s1, Figure S1: Phylogenetic analysis of CrzRs with RPCHR and ACPR. The following sequences were used: *Portunus trituberculatus* CrzR (PtCrzR, OL694706), *Rhodnius prolixus* CrzR1 and CrzR2 (RpCrzR1 and RpCrzR2, AND99324 and AND99325), *Carcinus maenas* CrzR and RPCHR (CmCrzR and CmRPCHR, AVA26879 and MF974387), *Daphnia pulex* RPCHR (DpRPCHR, ACD75498), *Ixodes scapularis* APCR1-4 (IsACPR1-4, AHB51763, AHB51764, AHB51765, AHB51766). *Tribolium castaneum* ACPR (TcACPR, NP001280549), *Nasonia vitripennis* ACPR and ACPRX1-X4 (NvACPR and NvACPRX1-4, NP001164571, XP031779949, XP031779950, XP031779951, XP031779952).
