# Supplementary figures and images for: Corazonin Stimulates Ecdysteroid Synthesis during the Molting Process of the Swimming Crab, Portunus trituberculatus
Source: Biology (Basel). 2024 Aug 18;13(8):630. doi: 10.3390/biology13080630 (PMC11352189; doi:10.3390/biology13080630)

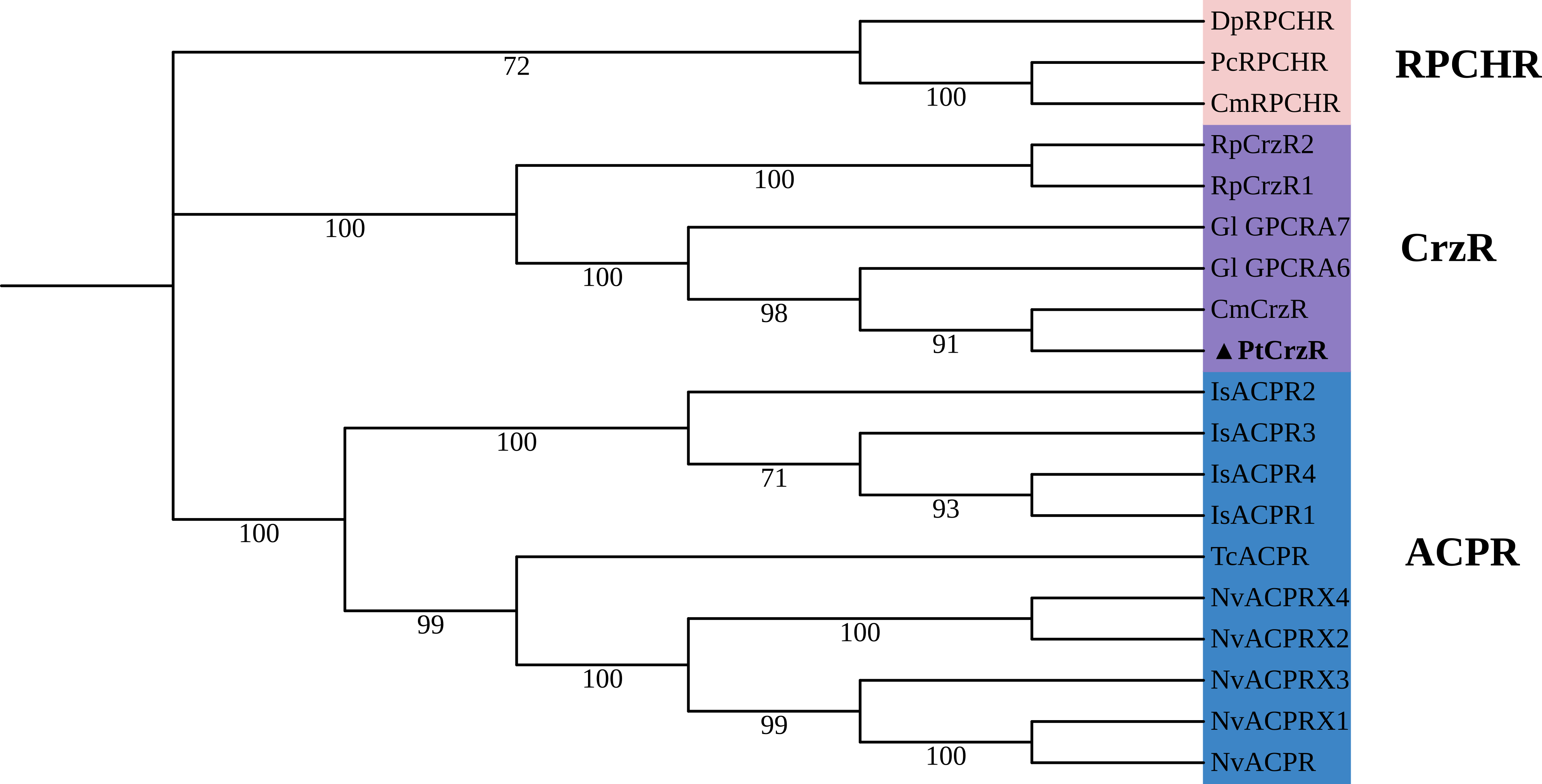

Supplement: Supplementary file 1 [file biology-13-00630-s001.zip › biology-3102509-supplementary.jpg]
